# Diagnostic value of endoscopic retrograde cholangiopancreatography and therapeutic value of endoscopic sphincterotomy in dogs with suspected hepatobiliary disorders

**DOI:** 10.1186/s12917-022-03241-4

**Published:** 2022-04-21

**Authors:** Vahideh Rahmani, Thomas Spillmann, Jorma Halttunen, Pernilla Syrjä, Mirja Ruohoniemi

**Affiliations:** 1 Department of Equine and Small Animal Medicine, Faculty of Veterinary Medicine, University of Helsinki, Helsinki, Finland; 2 Department of Gastrointestinal and General Surgery, Helsinki University Central Hospital, Helsinki, Finland; 3 Department of Veterinary Biosciences, Faculty of Veterinary Medicine, University of Helsinki, Helsinki, Finland

**Keywords:** Endoscopic retrograde cholangiopancreatography, ERCP, Endoscopic sphincterotomy, EST, Hepatobiliary disorders, Fluoroscopy, Gastroduodenoscopy

## Abstract

**Background:**

Conventional diagnostic methods have some limitations in diagnosing specific causes of canine hepatobiliary disorders. In the evaluation of the hepatobiliary system in dogs, ultrasonography (US) is the first imaging method of choice. Nonetheless, endoscopic retrograde cholangiopancreatography (ERCP) has also been proven to be a practicable technique for evaluating canine hepatobiliary (endoscopic retrograde cholangiography, ERC) and pancreatic duct (endoscopic retrograde pancreatography, ERP) disorders, providing additional therapeutic options by sphincterotomy (EST). To date, the efficacy and safety of diagnostic and therapeutic ERCP has not been evaluated in veterinary medicine literature. The present study sought to report complications and outcomes of dogs undergoing ERCP and EST, and to assess the usefulness of diagnostic ERCP by comparing the findings of US, ERCP and histopathological findings in liver and pancreas.

**Results:**

This retrospective case series comprises data collected from 15 dogs that underwent successful ERC/ERCP. Nine dogs underwent EST following ERC. US and ERC were best in agreement when assessing the common bile duct. In case of disagreement between the modalities, the ERC findings of the ductal structures were in line with the available pathology findings more often than the US findings, whereas the opposite was noted for the gallbladder. The technical success rates were 88.2% for ERC, 66.7% for ERP, and 81.8% for EST, with no major complications during or immediately after the procedure. Immediate bile flow after EST was recorded in 7/9 dogs but only four showed coinciding clinical and laboratory improvement and four dogs were euthanized within 1-6 days after EST.

**Conclusions:**

US remains a valuable initial diagnostic imaging method for hepatobiliary disorders and allows good assessment of the gallbladder. ERC can serve as a complementary procedure for diagnostic assessment of the hepatobiliary duct disorders. However, in order to improve the outcomes of EST, careful selection of patients for the procedure would require more advanced diagnostic imaging of the hepatobiliary area.

## Background

Conventional diagnostic procedures including history, physical examination and liver biochemical profile have some limitations in diagnosing specific causes of disorders of the canine hepatobiliary system and exocrine pancreas. In cases of hepatobiliary diseases, organ biopsies are required for histologic verification, but liver biopsy is an inherently invasive procedure that carries risk for some complications [1, 2]. Regarding chronic pancreatic diseases, the diagnosis often remains tentative since pancreatic biopsies are rarely taken for histologic confirmation and differentiation [3].

Diagnostic imaging of pancreaticobiliary diseases includes various techniques that are selected based on clinical signs, the diagnostic information sought, the technique’s invasiveness and the therapeutic needs of each individual case. Ultrasonography (US) is the most commonly used non-invasive imaging modality in the evaluation of the hepatobiliary system and the exocrine pancreas in dogs, offering high sensitivity but often lacking specificity, especially in patients with biliary and pancreatic ductal problems [4]. Endoscopic retrograde cholangiopancreatography (ERCP) has been proven to be an applicable, direct technique for evaluating hepatobiliary and pancreatic disorders both in human and veterinary medicine [5–10]. It is a minimally invasive technique, which combines endoscopy and fluoroscopy to image the biliary system (endoscopic retrograde cholangiography, ERC) and pancreatic duct (endoscopic retrograde pancreatography, ERP). In children, abnormal liver chemistries and suspected or established choledocholithiasis, follow-up for a biliary stricture, biliary or pancreatic duct leak of cholangitis, chronic pancreatitis, icterus, and abdominal pain have been listed as indications for ERCP [11]. With the introduction of endoscopic sphincterotomy (EST), therapeutic ERCP was subsequently developed [12]. Compared to US, ERCP requires general anesthesia, and complications such as pancreatitis, cholangitis, cholecystitis, bleeding and duodenal perforation have been reported in people [13–15]. A shift towards using ERCP for treatment purposes has emerged in human medicine together with a wider use of magnetic resonance cholangiopancreatography (MRCP) and endoscopic ultrasound [16].

In veterinary medicine, the feasibility of diagnostic ERCP in dogs with and without chronic gastrointestinal disorders [5, 9] and in healthy cats [8] has been demonstrated. In 2015, Berent et al. reported successful endoscopic biliary stenting in 5/7 healthy dogs and in 1/2 dogs with extrahepatic bile duct obstruction [10]. However, the efficacy and safety of diagnostic and therapeutic ERCP has not been evaluated in the veterinary literature to date. The primary aim of the present retrospective study was to characterize clinical, laboratory, ultrasonographic and hepatopancreatic histopathological findings, and to report indications, complications and outcome of dogs undergoing ERC/ERCP for diagnosis and EST for treatment. The secondary aim was to compare ultrasonographic and ERC/ERCP findings for assessing the usefulness of diagnostic ERC/ERCP in comparison to abdominal ultrasound.

## Results

### Dogs

Altogether 17 dogs underwent ERC or ERC with concurrent ERP between November 2007 and March 2019. The major papilla was identified in 17 and the minor papilla in 9 of the 17 dogs. ERC without the pancreatic component was performed successfully on 15 of the 17 dogs (88.2%) and concurrent ERP on 6 of 9 dogs (66.7%). Cannulation of the major papilla had failed in one small-sized dog (4.7 kg) and another dog had a mass at the major papilla, which prevented the insertion of the catheter. Thus, 15 dogs met the inclusion criteria.

At the time of ERC/ERCP, the dogs had a mean age of 6.8 ± 2.9 years and a mean body weight of 17.1 ± 8 kg. Of the 15 dogs, 8 were male (1 neutered) and 7 were female (4 spayed). The included 15 dogs represented 14 breeds (Table 1).

**Table 1:** Signalment and serum parameters of 15 dogs before undergoing ERC/ ERCP

Case	Breed	Age (Years)	Weight (Kg)	Clinical signs	Laboratory findings (reference range)			
					Bilirubin (2.5–8.5 μmol/l)	ALP (33–215 U/l)	ALT (18–77 U/l)	cPL (< 200 μg/l)
Dog 1^a^	Spanish Water Dog	8	18.9	Icterus, Vomiting	**786.4**	**1760**	**1574**	NP
Dog 2^a^	Red Irish Setter	9	24.8	Icterus, Vomiting, Diarrhea	**411.2**	**16,048**	**1089**	NP
Dog 3^a^	Giant Poodle	7	18.7	Icterus, Vomiting, Inappetence	**367.2**	**2318**	**1998**	**496**
Dog 4^a^	Nova Scotia Duck Tolling Retriever	6	24	Icterus, Vomiting, Diarrhea	**183.4**	**5833**	**2870**	103
Dog 5^a^	Bichon Frise	6	6.7	Icterus, Vomiting	**176.6**	**6759**	**642**	NP
Dog 6^a^	Parson Russell Terrier	11	9	Icterus, Vomiting, Inappetence	**51.6**	**16,442**	**2434**	79
Dog 7^a^	White Shepherd Dog	8	27.7	Icterus, Vomiting, Inappetence, Diarrhea, Weight loss	**49.5**	**5049**	**2369**	35
Dog 8^a^	Nova Scotia Duck Tolling Retriever	6	18.5	Icterus, Vomiting, Inappetence	**45.2**	**1051**	**1837**	**267**
Dog 9^a^	French Bulldog	3	6.9	Icterus, Weight loss	**14**	**11,429**	**850**	**217**
Dog 10	Belgian Shepherd Dog	2	29	Vomiting, Abdominal pain, Weight loss	4.3	196	23	NP
Dog 11	Jack Russel Terrier	3	10	Vomiting, Abdominal pain	4.3	102	42	**1000**
Dog 12	Shetland Sheepdog	8	5.5	Vomiting, Abdominal pain	3.9	76	77	NP
Dog 13	Short-haired Collie	11	24.8	Vomiting, Borborygmus	3.3	173	**637**	**694**
Dog 14	Fox Terrier	11	13	Vomiting, Diarrhea, Abdominal pain	2.6	**310**	32	–
Dog 15	Finnish Lapphund	4	20	Vomiting	2.5	151	39	118

Abnormal results in bold font

*ALP* Alkaline phosphatase, *ALT* Alanine aminotransferase, *cPL* canine pancreatic lipase, *NP* not performed

^a^icteric

### Clinical signs

Dogs were presented with one or more of the following gastrointestinal signs ordered by their frequency of appearance: chronic or recurrent vomiting (14/15), chronic or recurrent diarrhea (4/15), recurrent abdominal pain (4/15), recurrent inappetence (4/15), weight loss (3/15), and borborygmus (1/15) (outlined in Table 1). A sign of > 3 weeks duration was considered chronic. Nine of the 15 dogs had icterus at the time of the presentation.

### Laboratory parameters before ERC/ERCP

A complete serum biochemical panel before ERC/ERCP was available for all dogs. Deviations from the reference range were mainly seen for the serum parameters of the liver (Table 1). Hyperbilirubinemia was present in 9/15 dogs (60%) with median serum bilirubin of 176.6 μmol/l (range 14–786.4 μmol/l). All icteric dogs (Dogs 1–9) had concurrent elevated activities of alkaline phosphatase (ALP) and alanine aminotransferase (ALT). Overall, serum activity of ALP and ALT was elevated in 10/15 dogs and canine pancreatic lipase (Spec cPL) was elevated in 5/9 dogs (534.8 ± 322.4 μg/l, Table 1). Coagulation tests were performed for 9/11 dogs before EST, but no abnormalities were found.

### Abdominal ultrasonography

The findings of abdominal US of the liver parenchyma, the biliary system and the pancreas were grouped in icteric and non-icteric dogs and are summarized in Table 2. The appearance of the major papilla was recorded only for five icteric and one non-icteric dogs. The pancreatic duct was not mentioned in any of the reports.

**Table 2:** Findings of 15 dogs in ultrasound, ERC (including endoscopy) and ERP

Case	Liver	Major papilla		Gallbladder		Common bile duct		Extrahepatic duct	Intrahepatic duct	Pancreatic area	
	US	US	ERC (Endoscopy)	US	ERC	US	ERC	ERC	ERC	US (pancreas)	ERP (ducts)
Dog 1^a^	Hypo-echoic	Normal	Prominent (open)	Normal	Normal	Normal	Dilated 20 mm and narrowed	Normal	Normal	Hyperechoic	NP
Dog 2^a^	Hyper-echoic	Prominent	Normal	Distended, debris	No filling	Dilated 10 mm	No filling (Obstruction)	Dilated	Normal	Hyperechoic	Normal
Dog 3^a^	Hyper-echoic	Normal	Normal	Distended, debris	NR	Dilated 10 mm	Dilated 5 mm (Obstruction)	NR	Dilated	Normal	Normal
Dog 4^a^	Hyper-echoic	NR	Normal	Normal	Normal	Normal	Normal	Normal	No filling	Normal	Normal
Dog 5^a^	Hyper-echoic	NR	Normal	Distended, debris	Normal	Normal	Narrowed	Dilated 5 mm	Normal	Hypoechoic	NP
Dog 6^a^	Hetero-genic	Prominent	Prominent	Distended, debris	NR	Dilated 4.5 mm	Dilated	Dilated	NR	Heterogenous	NP
Dog 7^a^	Hyper-echoic	NR	Normal	Distended, debris	No filling	Dilated 5.3 mm	No filling	Dilated 10 mm	NR	Hyperechoic	NP
Dog 8^a^	Hyper-echoic	Prominent	Prominent	Distended, debris	NR	Dilated9 mm	Dilated 10 mm	NR	NR	Normal	NP
Dog 9^a^	Hypo-echoic	NR	Normal	Normal	Normal	Normal	Normal	Normal	Normal	Hyperechoic	Normal
Dog 10	Normal	NR	Normal	Normal	Normal	Normal	Abnormal course	NR	NR	Normal	NP
Dog 11	Normal	NR	Stenosis	Normal	Normal	Normal	Normal	Normal	NR	Hypoechoic	Normal
Dog 12	Normal	NR	Very small	Distended, mucocele	NR	Normal	Normal	Normal	Normal	Hyperechoic	NP
Dog 13	Normal	NR	Stenosis	Distended, debris	Normal	Dilated 10 mm	Dilated 10 mm	NR	NR	Normal	NP
Dog 14	Normal	Prominent	Normal	Distended, debris	Normal	Normal	Normal	Normal	Decreased filling	Hyperechoic	Normal
Dog 15	Normal	NR	Normal	Normal	Normal	Normal	Normal	NR	NR	Hypoechoic	Abnormal course

*US* ultrasonography, *ERC* endoscopic retrograde cholangiography, *ERP* endoscopic retrograde pancreatography, *NR* not reported, *NP* not performed

^a^icteric

All icteric dogs showed changes in the echogenicity of the liver parenchyma. In one dog (Dog 6), the liver appeared heterogenous with multiple hypo- and hyperechoic nodules. In three dogs, the major papilla was prominent. Gallbladder abnormalities were seen in 6/9 dogs with mild-to-moderate echogenic debris, associated with hyperechoic content attached to the gallbladder wall in one dog (Dog 3). The Common bile duct (CBD) was dilated in five dogs. There was no mention of the extrahepatic ducts, but two dilated intrahepatic ducts were reported in one dog (Dog 5). The echogenicity of the pancreas showed abnormal changes in 6/9 dogs.

**Fig. 1:**
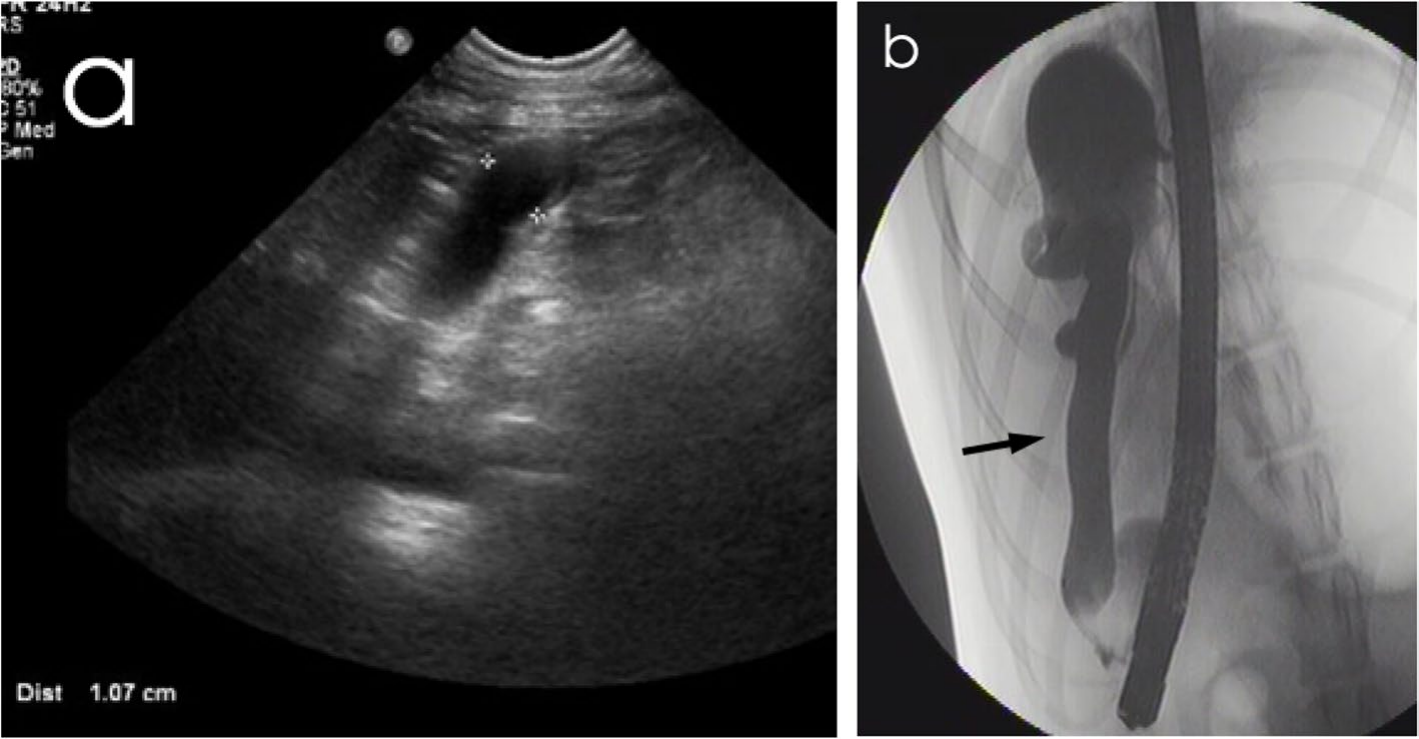
Images of a non-icteric 11-year-old male Short-haired Collie (Dog 13). **a** Ultrasonographic image acquired before ERC, showing dilatation of the common bile duct (cursors). **b** Fluoroscopic image acquired during ERC, representing dilatation of the common bile duct (arrow, maximum diameter 10 mm) and the gallbladder filled with the contrast material

All six non-icteric dogs had normal hepatic parenchyma in US and a prominent major papilla was reported in one dog (Dog 14). Two dogs had mild-to-moderate gallbladder debris, one dog a mucocele (Dog 12) and one dog (Dog 14) showed a concurrent hyperechoic focus (40 mm) with acoustic shadowing inside the gallbladder. The CBD was dilated in one dog (Dog 13) (Fig. 1). No ultrasonographic findings were reported for the extra- and intrahepatic ducts. The echogenicity of the pancreas showed abnormal changes in 4/6 dogs.

### ERC/ERCP indications and findings

The indications for ERC/ERCP in the 15 dogs are shown in Table 3. More than half of the dogs (9 dogs) showed five to seven indications for performing ERC/ERCP and six dogs revealed one to four indications. The most common indications were chronic gastrointestinal signs (15/15 dogs), followed by elevated liver enzymes (11/15 dogs), abnormal US findings in the pancreatic area (10/15 dogs), abnormal US findings in the biliary system (9/15 dogs), jaundice (9/15 dogs), abnormal US findings in the liver (9/15 dogs), elevated cPL (5/15 dogs), and abnormal US findings in the major papilla (4/15 dogs).

**Table 3:** Indications for diagnostic ERC/ERCP in 15 dogs

Case	Indication								Total number of indications in the dog
	Chronic gastro-intestinal signs	Laboratory			Ultrasound				
		Icteric (hyper-bilirubinemia)	Elevated liver enzymes	Elevated cPL	Abnormality in the major papilla	Abnormality in the liver	Abnormality in biliary system	Abnormality in the pancreatic area	
Dog 1^a^	+	+	+	–	–	+	–	+	5
Dog 2^a^	+	+	+	–	+	+	+	+	7
Dog 3^a^	+	+	+	+	–	+	+	–	6
Dog 4^a^	+	+	+	–	NR	+	–	–	4
Dog 5^a^	+	+	+	–	NR	+	+	+	6
Dog 6^a^	+	+	+	–	+	+	+	+	7
Dog 7^a^	+	+	+	–	NR	+	+	+	6
Dog 8^a^	+	+	+	+	+	+	+	–	7
Dog 9^a^	+	+	+	+	NR	+	–	+	6
Dog 10	+	–	–	–	NR	–	–	–	1
Dog 11	+	–	–	+	NR	–	–	+	3
Dog 12	+	–	–	–	NR	–	+	+	3
Dog 13	+	–	+	+	NR	–	+	–	4
Dog 14	+	–	+	–	+	–	+	+	5
Dog 15	+	–	–	–	NR	–	–	+	2

+: present; −: not present

*NR* not reported

^a^icteric

The ERC/ERCP findings of the dogs are reported in Table 2.

**Fig. 2:**
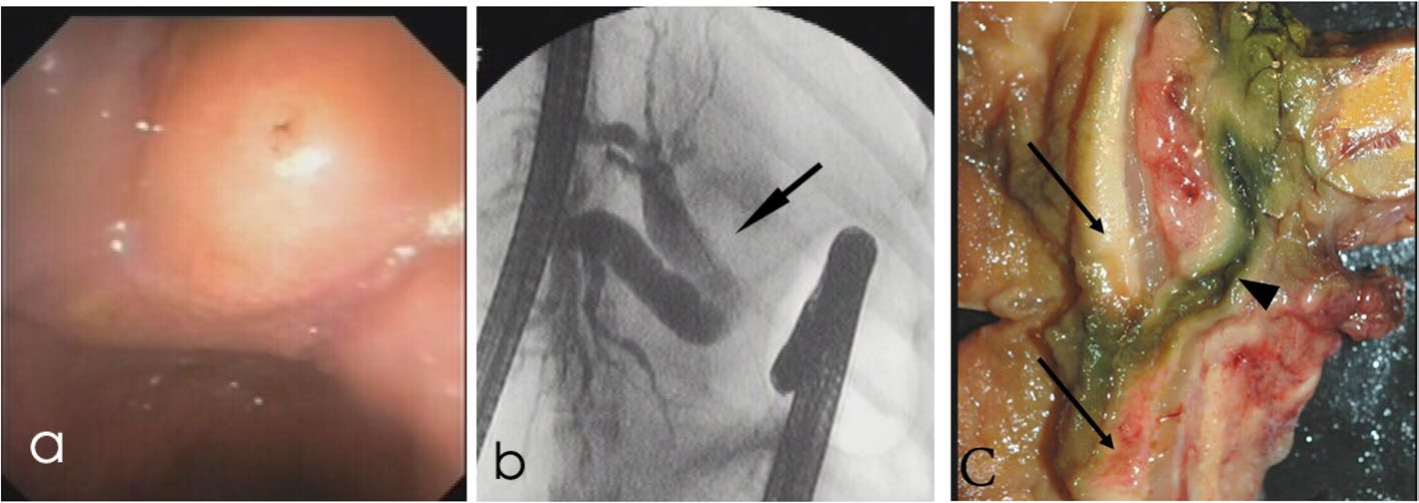
Images of an icteric 9-year-old female Red Irish Setter (Dog 2). **a** Endoscopic image of the prominent major papilla. **b** Fluoroscopic image acquired during ERC showing marked dilatation of the extrahepatic ducts (arrow) without contrast filling of the common bile duct. **c** Thickened duodenal wall due to Infiltrative carcinoma (arrows) around the common bile duct (arrowhead) at autopsy 1 day after ERC and EST

**Fig. 3:**
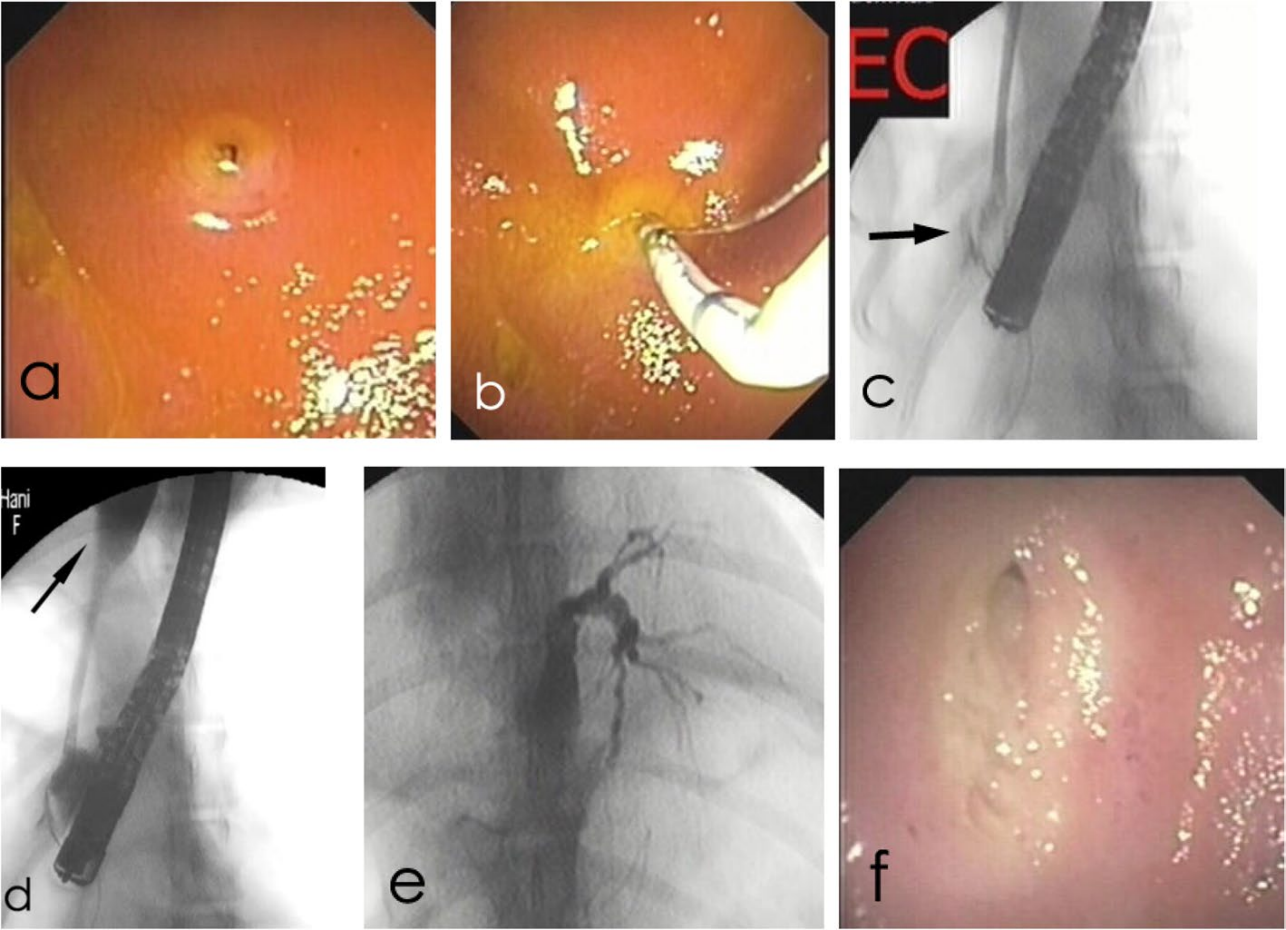
Images of an icteric 7-year-old female Giant Poodle (Dog 3). **a** Endoscopic image of the major papilla before EST. **b** Endoscopic image while the sphincterotomy catheter is entering the major papilla. **c** Fluoroscopic image acquired during ERC, showing radiolucent concretions (arrow) at the beginning of the common bile duct. **d** Fluoroscopic image showing dilatation of the common bile duct (arrow, maximum diameter 5 mm). **e** Fluoroscopic image representing marked dilatation of the intrahepatic duct. **f** Endoscopic image of the major papilla 3 weeks after EST

Conventional endoscopy of the nine icteric dogs revealed a prominent major papilla in three dogs and a prominent minor papilla in one dog (Dog 2). In ERC, the CBD was considered dilated in 4/9 dogs and narrowed in 2/9 dogs. In one dog (Dog 2), the gallbladder and CBD did not fill with contrast (Fig. 2). The gallbladder and CBD were not assessable in another dog (Dog 7), since the contrast medium leaked out of the major papilla to the duodenum during the procedure. Four dogs (Dogs 2, 5, 6, 7) had dilation of the extrahepatic bile ducts and in one dog (Dog 3) dilated intrahepatic bile ducts were visible (Fig. 3). In Dog 4, no contrast reached the intrahepatic bile ducts even after several attempts with repositioning of the catheter. ERP was performed in 4/9 icteric dogs, and all of them showed normal findings of the pancreatic ducts.

**Fig. 4:**
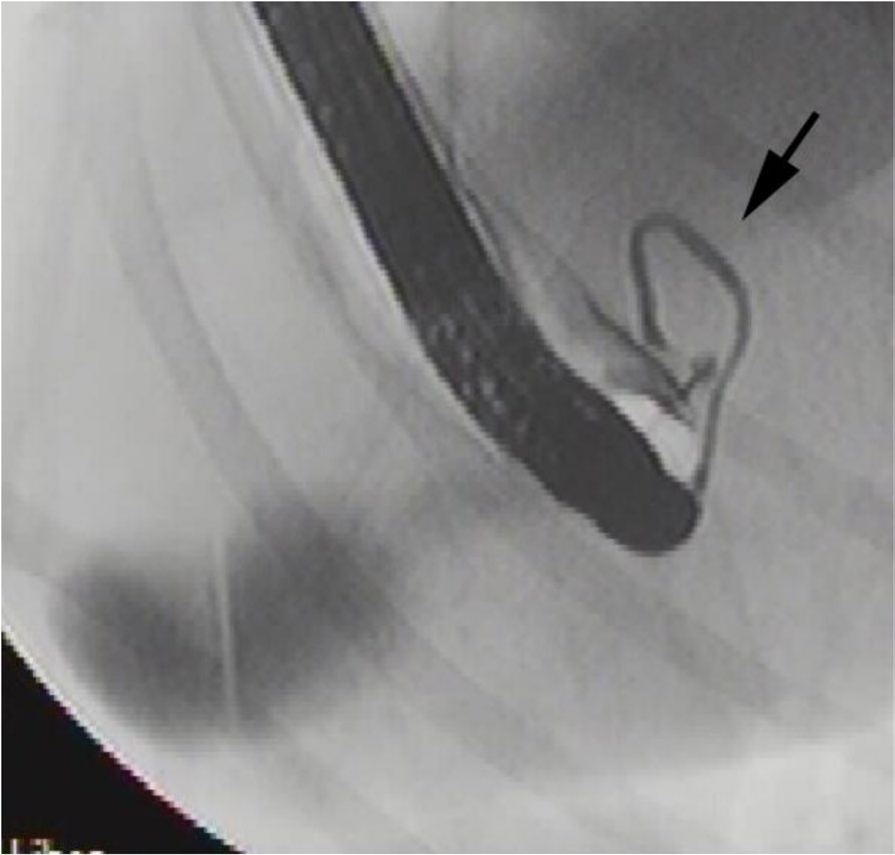
Fluoroscopic image of a non-icteric 2-year-old male Belgian shepherd dog (Dog 10) acquired during ERC representing abnormal course of the CBD (arrow) in a half circle around the duodenum and not straight to the gallbladder

Conventional endoscopy of the six non-icteric dogs revealed an abnormal major papilla in three dogs. The minor papilla was endoscopically normal in five dogs and one dog did not have any endoscopic report of the minor papilla. ERC revealed a dilated CBD in one dog (Dog 13) (Fig. 1), and in one dog (Dog 10) the CBD was found to course in a half circle around the duodenum and not straight to the gallbladder (Fig. 4). Extrahepatic ducts were normal in all three non-icteric dogs having reports in ERC. One dog had decreased filling of the intrahepatic duct (Dog 14). ERP was performed in 3/6 non-icteric dogs and one dog revealed abnormal course of duodenal branch of the pancreatic duct, going cranial to the diaphragm (Dog 15).

### Comparison of ultrasonographic, ERC/ERCP and histopathology findings

When comparing ultrasonographic and ERC/ERCP findings with each other, and where possible with findings of histopathology of the samples taken at endoscopy for ERC/ERCP, laparoscopy, laparotomy or autopsy (Table 4), the following agreements and disagreements were seen concerning the major papilla, gallbladder, CBD, extrahepatic ducts, intrahepatic ducts and pancreatic duct. Reports of both US and endoscopy for ERC for the major papilla were available for 6/15 dogs. There was full agreement on a normal papilla in one dog (Dog 3) and on a prominent papilla in two dogs (Dogs 6 and 8). In three dogs with disagreements between US and endoscopy, no morphologic examination of the papilla was possible for final assessment.

**Table 4:** Histopathologic findings of the liver, pancreas, and duodenum of 15 dogs that underwent ERC/ERCP and/or EST

Dog	Type of sampling	Hepatobiliary system	Pancreas	Duodenum
Dog 1^a^	Biopsy Laparoscopy	Moderate chronic neutrophilic cholangiohepatitis, Biliary stasis	Focal severe fibrosing pancreatitis with multifocal moderate peripancreatic chronic histiocytic steatitis	Not sampled
Dog 2^a^	Autopsy	Moderate chronic neutrophilic cholangiohepatits, Moderate biliary stasis, Pancreatic carcinoma metastasis in the liver	Exocrine pancreatic carcinoma	Infiltration of pancreatic carcinoma into the duodenal wall compressing the common bile duct
Dog 3^a^	Biopsy Endoscopy	Not sampled	Not sampled	Moderate chronic diffuse lymphoplasmacytic enteritis
Dog 4^a^	Biopsy Laparoscopy	Severe chronic neutrophilic and fibrosing cholangiohepatitis	Not sampled	Not sampled
Dog 5^a^	Biopsy Laparotomy	Severe diffuse chronic hepatic lipidosis and multifocal extramedullary hematopoiesis	No significant parenchymal changes, Mild interstitial fibrosis, Acute endothelial swelling	Not sampled
Dog 6^a^	Biopsy Laparoscopy	Moderate multifocal chronic neutrophilic and fibrosing hepatitis, Moderate chronic lymphoplasmacytic cholecystitis with mild cystic mucinous hyperplasia of gallbladder mucosa	Not sampled	Not sampled
Dog 7^a^	Autopsy	Moderate chronic neutrophilic and fibrosing cholangiohepatitis, Moderate cystic mucinous hyperplasia of gallbladder mucosa	No significant histological changes	No significant histological changes
Dog 8^a^	Autopsy	Moderate chronic fibrosing and neutrophilic cholangiohepatits	No significant histological changes	Local severe chronic lymphoplasmacytic mural enteritis at papilla
Dog 9^a^	Biopsy Laparoscopy	Moderate chronic fibrosing and neutrophilic cholangiohepatits (Autopsy: Focal abscess and severe biliary stasis)	Not sampled	No significant histological changes
Dog 10	Autopsy	Moderate multifocal fibrosing and histiocytic inflammation involving areas around duodenum, pancreas and a curved common bile duct, Hepatic biliary stasis	Severe peripancreatic chronic histiocytic steatitis	No significant histological changes
Dog 11	Biopsy Endoscopy	Not sampled	Not sampled	Mild chronic diffuse eosinophilic enteritis
Dog 12	_	Not sampled	Not sampled	Not sampled
Dog 13	Biopsy Endoscopy	Not sampled	Not sampled	Mild chronic diffuse lymphoplasmacytic enteritis with local moderate acute fibrinonecrotizing duodenitis
Dog 14	Autopsy	Mild hepatic fibrosis, Mild acute necrotizing cholecystitis	Acute severe multifocal necrotizing pancreatitis	Local severe necrotizing peritonitis, Moderate diffuse chronic lymphoplasmacytic enteritis
Dog 15	Biopsy Endoscopy	Not sampled	Not sampled	Mild diffuse chronic lymphoplasmacytic enteritis

^a^Icteric

Reports of both modalities for gallbladder were available for 11/15 dogs. There was full agreement on a normal gallbladder in six dogs (Dogs 1, 4, 9, 10, 11, 15). From five dogs with different US and ERC findings, Dogs 2 and 7 showed a distended gallbladder with debris in US but no contrast filling of the gallbladder in ERC. Histopathology in Dog 2 revealed biliary stasis, and in Dog 7 cystic mucinous hyperplasia of the gallbladder mucosa. Dogs 5, 13 and 14 had distended gallbladder with debris in US, but normal gallbladder in ERC. Laparotomy of Dog 5 revealed a big and pink gallbladder, which was easily emptied when manually pressed. Histopathology showed necrotizing cholecystitis in Dog 14.

Both US and ERC reports of the CBD were available for all 15 dogs. Agreement on a normal CBD occurred in six dogs (Dogs 4, 9, 11, 12, 14, 15) and on a dilated CBD in four dogs (Dogs 3, 6, 8, 13). From five dogs with disagreement between US and ERC, Dogs 1, 5 and 10 had a normal CBD in US but ERC reported dilation in Dog 1, narrowing in Dog 5 and an abnormal course in Dog 10. Histopathology revealed chronic cholangiohepatitis and biliary stasis in Dog 1 and focal granulomatous peritonitis in Dog 10 involving the CBD, pancreas and intestine together with a torturous course of the duct. In Dog 5, laparotomy showed a dilated CBD with no stricture. Dogs 2 and 7 had a dilated CBD in US, but no contrast filling was visible in ERC. Histopathology showed pancreatic carcinoma with infiltration into the duodenal wall around the CBD (Fig. 2), chronic cholangiohepatitis with carcinoma metastasis and biliary stasis in Dog 2, and chronic cholangiohepatitis in Dog 7.

Extrahepatic bile ducts were not mentioned in any of the US reports. ERC reports for extrahepatic bile ducts were available in 10 dogs (normal in Dogs 1, 4, 9, 11, 12, 14; dilated in Dogs 2, 5, 6, 7). Histopathology in the four dogs with dilated extrahepatic duct in ERC revealed biliary stasis in Dog 2, due to a pancreatic carcinoma infiltrating the duodenal wall and compressing the CBD, hepatic lipidosis without lesions targeting the biliary tract in Dog 5, chronic cholecystitis with cystic mucinous hyperplasia of the gallbladder mucosa in Dog 6, and chronic cholangiohepatitis and cystic mucinous hyperplasia of the gallbladder mucosa in Dog 7. In one dog (Dog 5), two dilated intrahepatic ducts were reported in US. However, ERC of the same dog revealed a 2 cm long stricture in the CBD starting from the major papilla and dilated extrahepatic ducts were reported. Laparotomy revealed no obvious cause of the suspected CBD stricture. Histopathology of the liver parenchyma biopsies revealed severe lipidosis but no significant lesions in the intrahepatic ducts.

The pancreatic duct was not mentioned in any of the US reports. ERP reports were available for seven dogs (normal in Dogs 2, 3, 4, 9, 11, 14; abnormal course in Dog 15). Autopsy reports of the pancreas were available for Dog 2 directly after ERC and Dog 14 at day 178 after ERC. Histopathology showed metastasizing exocrine pancreas carcinoma in Dog 2 and acute sever multifocal necrotizing pancreatitis in Dog 14.

### EST

EST was attempted in 11/15 dogs. It was successful in 6/7 icteric dogs (Dogs 2, 3, 5, 6, 7, 8) but not in Dog 1. In the non-icteric dogs, EST was successful in 3/4 dogs (Dogs 12-14) but in one dog (Dog 11) the procedure was performed twice. The first attempt led to local bleeding, but the second attempt 4 weeks later was successful. The indication for EST included one or more of the following ERC findings: dilated CBD (5 dogs), prominent major papilla (3 dogs), stenosis or small major papilla (3 dogs), narrowed CBD (2 dogs), no contrast filling of the CBD (2 dogs), and obstruction of the CBD (2 dogs). The procedure was successful in 9/11 dogs (81.8%), of which seven revealed bile flow immediately after cutting the major papilla, whereas no bile flow occurred in two (Dogs 2 and 7).

### ERC/ERCP and EST complications

None of the dogs that underwent ERC/ERCP or EST developed procedure-related severe complications or deaths. Minor complications occurred in two dogs. In one (Dog 1), the tip of the endoscope broke while cannulating for EST. However, all parts of the endoscope were removed, and the procedure ended up in a successful diagnostic ERC. In the other (Dog 11), it was difficult to cannulate the major papilla with the sphincterotome at the first attempt of performing EST. Some opening of the papilla was possible, but the procedure was stopped due to strong intestinal peristalsis, development of bleeding and an intramucosal injection of contrast medium. Four weeks later, the EST procedure was repeated and was successful with opening of the major papilla.

### Outcome after EST

**Fig. 5:**
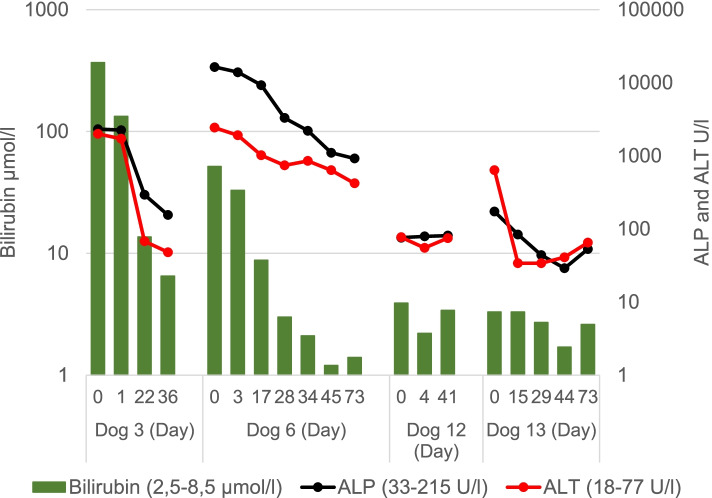
Serum bilirubin concentrations and serum activities of alkaline phosphatase (ALP) and alanine aminiotransferase (ALT) of four dogs before and after successful EST

Improvement in the clinical status and the laboratory parameters was noted in 1/6 of the icteric dogs (Dog 3) and 2/4 of the non-icteric dogs (Dogs 12 and 13). The elevated serum liver parameters returned to the normal level in all these dogs shortly after EST (Fig. 5). In Dog 3, US showed that the diameter of the CBD decreased from 10 mm to 4 mm in 1 day after the procedure. The dog remained clinically unremarkable until lost for follow up 71 days after EST. Dog 12, with a gallbladder mucocele, had short-term clinical improvement after EST, but episodes of recurrent abdominal pain returned. The dog remained free of clinical signs throughout the follow up when permanently receiving ursodeoxycholic acid. Four months after EST, US showed a normal pancreas and the appearance of the mucocele was unchanged until the last US examination performed 509 days after EST. Long-term follow up of Dog 13 revealed development of septic peritonitis 306 days after EST and the dog was euthanized on owner’s request. Since the owner refused an autopsy the cause of septic peritonitis remained open.

Dog 6 showed some clinical improvement after EST, and in the ultrasonographic examination 2 weeks later the gallbladder, CBD and major papilla were reported as normal. However, although the markedly elevated serum liver enzymes (ALP and ALP) gradually decreased, they were still above normal level 2 months after EST (Fig. 5) and the dog had repeated phases of fever and abdominal pain being responsive to metronidazole and enrofloxacin. Four months after EST, the dog underwent laparoscopy with liver biopsy and bile aspiration. Histopathology showed chronic hepatitis and chronic cholecystitis with hyperplasia of the gallbladder mucosa. Bacterial culture revealed extended spectrum beta-lactamase (ESBL) *E. coli* in the bile but was negative for the liver tissue sample. The gallbladder was removed surgically 12 days after laparoscopy and the infection with ESBL E.coli was confirmed in bile and feces samples. After the surgery, the dog recovered fully but the bacterial culture of the feces samples remained positive for ESBL E.coli. The dog was last presented 22 months after surgery having no relapse of fever and abdominal pain but still moderately elevated liver enzymes.

No improvement after EST was seen in 4/6 icteric (Dogs 2, 5, 7, 8) and in 2/4 non-icteric dogs (Dogs 11 and 14). The four icteric dogs were euthanized 1-6 days after the procedure. Dogs 2 and 7 showed no bile flow from the major papilla after EST and no clinical improvement immediately after the procedure, and the owners opted for euthanasia the next day. Autopsy revealed infiltrative exocrine pancreatic carcinoma in the duodenal wall suppressing the CBD in Dog 2 and cholangiohepatitis with cystic mucinous hyperplasia in the mucosa of the enlarged gallbladder in Dog 7. In Dog 8, the gallbladder and CBD remained dilated in US examination and serum bilirubin concentration was elevated for 1 day after EST. The owner refused surgical procedures and chose euthanasia 2 days after EST. Local chronic mural enteritis at the major papilla was diagnosed at autopsy. The fourth dog (Dog 5) was euthanized 6 days after EST because of its poor clinical condition. It was impossible to establish the cause of the CBD stricture since the owner declined autopsy. Of the non-icteric dogs, Dog 11 with chronic eosinophilic enteropathy continued to have recurrent severe abdominal pain and increased Spec cPL concentrations as signs of chronic pancreatitis. The dog did not respond appropriately to conventional treatment measures and was euthanized 2 months after EST. The owners denied necropsy. The other non-icteric dog (Dog 14) lacked follow-up reports shortly after EST, however, 6 months after EST the dog showed acute diarrhea, vomiting and abdominal pain, coupled with laboratory findings of elevated ALP, ALT and cPL values. A hyperechoic pancreas was visible in US. Exploratory laparotomy revealed gross alterations in the pancreas, and the owner opted for euthanasia and autopsy, which revealed severe acute necrotizing pancreatitis and peripancreatic peritonitis.

## Discussion

Chronic gastrointestinal signs associated with elevated liver enzymes, abnormal US findings in the pancreatic area or in the biliary system and/or icterus were the most common indications for conducting ERC/ERCP in dogs included in the present retrospective study. Similarly, ERCP has been used for evaluation and management of biliary and pancreatic disorders in children. The most common indications for ERCP in children have been reported to be abnormal liver chemistries and suspected or established choledocholithiasis, together accounting for 50% of indications in a large retrospective study [11] Other reported pediatric indications included follow-up for a biliary stricture, biliary or pancreatic duct leak of cholangitis, chronic pancreatitis, icterus, and abdominal pain [11]. In human medicine, ERCP has been used also for emergency procedures, with indications such as acute cholangitis, acute pancreatitis, and post-operative bile leak [17]. Until today, the use of ERCP in veterinary medicine has been limited to chronic cases, mainly due to the demanding technique of the procedure.

All 15 dogs of this study revealed some abnormal findings in US and ERC/ERCP examinations. The best agreement between US and ERC occurred for assessing the normality or abnormality of the CBD. In case of disagreement of both modalities, the ERC findings of the ductal structures were in line with the available laparotomy, laparoscopy, or autopsy findings more often than the US findings. In contrary, for the gallbladder the US findings were closer to the laparotomy, laparoscopy, or autopsy findings. In a human study, US was in agreement with abnormal ERCP findings in 101 of 120 patients (sensitivity 84%) and of normal ERCP findings in 70 of 73 patients (specificity 95%) [18]. US has been shown to be more sensitive in diagnosis of dilated or strictured bile ducts than finding choledocholithiasis [18]. ERCP is considered complimentary to US specifically when the problem is located within the pancreatobiliary ductal anatomy.

The comparison of US and ERC results revealed different degrees in agreement and disagreement dependent on the reported anatomical structure. The major papilla was visible in all 15 dogs scanned by endoscopy for ERC examination, but was reported only in six dogs by using US. This is in agreement with a previous study, which found that the major papilla was visualized ultrasonographically in 42% of dogs without evidence of abdominal disorders [19]. The visibility was negatively influenced by the presence of air or food in the gastrointestinal tract and increased body weight [19]. US and endoscopy for ERC/ERCP disagreed concerning the major papilla in three dogs of our study, which may be due to contractile activity of the sphincter of Oddi [20] or different types of the major papilla [21]. Since no histopathology reports were available for the major papilla in these three dogs, however, it is impossible to show which modality had the correct diagnosis.

US and ERC agreed in a normal or dilated CBD in 10/15 dogs. A recent study showed that US is a useful modality for detection of the canine CBD diameter and that US results were not significantly different from those of CT [22]. In human medicine, bile duct dilatation has been detected by US with the sensitivity of 85–95% for experienced examiners [23]. In all three dogs (Dogs 1, 5, 10) that showed normal CBD in US but abnormal in ERC, pathology revealed abnormal findings of the hepatobiliary system which highlights the fact that a normal US examination cannot rule out hepatic or ductal pathology and the possible need for ERC. Two dogs (Dogs 2 and 7) had a dilated CBD in US but the duct did not fill with contrast material in ERC. In Dog 2, this was found to be due to infiltration of carcinoma within the intestinal wall, causing pressure to the duct, and in the other dog, precipitated bile was detected within the gallbladder at autopsy. MRCP could be helpful in cases where space-occupying masses prevent contrast material visibility within the biliary ducts [4].

The gallbladder was visible and could be assessed more often by US than by ERC, and the US findings were more in agreement with the laparotomy, laparoscopy, or autopsy findings where available. It can be speculated that the diagnostic value of US is preferentially for the gallbladder, and ERC for ductal structures. In human medicine, it has been shown that US has high sensitivity for detecting cholecystitis [24]. Another study showed that CT was significantly more sensitive for diagnosing cholecystitis than US [25], but because of the speed and portability, US is still used as the initial imaging technique for evaluating patients with suspected acute cholecystitis.

Extrahepatic ducts were not mentioned in any of the US reports. Extrahepatic ducts are usually poorly visualized owing to overlying bowel gas and the normal intrahepatic ducts are not visible in US [26]. Dilated intrahepatic ducts were reported in one dog in US, but ERCP revealed an actual dilation of extrahepatic ducts. Since ERCP has the ability to show the entire biliary system in one view, it can be more reliable in localizing the abnormality.

The technical success rate of ERC and ERP was 88.2 and 66.7%, respectively. This is comparable with previously reported success rates of 67–75% for ERC in dogs and cats and of 70% for ERP in dogs [8–10]. Failures have been reported to be related to difficulties in the duodenal papilla cannulation in a small-sized dog, intraduodenal contents, changes of the duodenal mucosa, and decrease in gastric tone [9]. One possibility to improve the visibility of the minor papilla is the use of chromoendoscopy, as reported for cats [8].

In the six dogs that underwent ERP, the duodenal and gastric branches of the main pancreatic ducts were clearly visible with no signs of duct abnormalities in five dogs. In one dog (Dog 15), with serum cPL concentration in the reference range, the duodenal branch of the pancreatic duct did not go caudally but cranially to the diaphragm. In humans, abnormal contrast pancreatograms are used to grade the stage of chronic pancreatitis by the Cambridge classification system from mild to severe [27]. It remained open whether the ERP finding of the dog in our study was a variation of normality, an artifact, or a possible pathologic finding, since the dog underwent no direct examination of the pancreas by invasive diagnostic methods.

The success rate of EST was 81.8% with no major procedure-related complications during or immediately after the procedure**.** In humans, biliary EST is associated with several complications, both in the short- and the long-term [14, 15]. Due to the invasive nature of the procedure, these complications are inevitable and observed in some patients, depending on patient- and procedure-related factors [12]. The short-term complications of EST are bleeding, perforation, pancreatitis and cholangitis [14, 15]. Early identification and appropriate management of complications is essential to reduce mortality and morbidity. Of the known short-term complications of EST, only mild bleeding occurred in one dog of our study. In human medicine, acute pancreatitis is the most common post-ERCP complication, with an incidence of 2–10% [28]. One dog (Dog 14) showed acute severe necrotizing pancreatitis 6 months after EST, but there are no laboratory or clinical records of the dogs until 6 months after EST. It can be suggested that an acute pancreatitis developing 6 months after EST is unlikely to have been caused by the procedure.

Only four of nine dogs showed clinical and laboratory improvement after EST. This finding is similar to the findings of previous studies reporting clinical improvement after EST in a dog with papillary stenosis [9] and in one dog with signs of extrahepatic duct obstruction treated by EST and biliary stenting [10]. One dog (Dog 6), regardless of clinical and laboratory improvement after EST, developed recurrent fever 4 months after EST and bacterial culture revealed ESBL *E. coli* in the bile and feces. A study in humans showed that the prevalence of post-ERCP biliary tract infections was 4% of the patients [29]. It remained open whether the ESBL E.coli infection in Dog 6 was a complication of ERC or an ascending infection from intestine after EST.

The finding of dilated CBD in ERC without evidence of obstruction in two icteric dogs, was of interest. The cause of this dilation could not be identified. However, after EST there was marked bile flow into the duodenum. A report on six cats with sphincter of Oddi pathology speculated that patients without obvious pathology at the papilla might have sphincter dysfunction with extrahepatic biliary obstructions due to a chronic inflammatory enteropathy [30]. Histopathology of the duodenal biopsy samples in Dog 8 revealed mural enteritis at the papilla.

Four dogs underwent EST without improvement and were euthanized within days after the procedure. Clear causes of posthepatic and intrahepatic cholestasis were established by autopsy in three dogs. Dog 2 had an exocrine pancreatic carcinoma infiltrating the duodenal wall, Dog 7 a moderate chronic cholangiohepatitis and cystic mucinous hyperplasia of the gallbladder mucosa and Dog 8 moderate chronic cholangiohepatitis and severe chronic mural enteritis. No clear cause was found for Dog 5 with a CBD stricture and increasing hyperbilirubinemia after EST, since hepatic lipidosis was the sole histopathological finding in the liver. Prolonged cholestasis after ERC may be one possible reason. It is a very rare but recognized complication following ERCP in humans [31]. The exact mechanism for prolonged jaundice post-ERCP has remained unclear but it has been suggested that it may be directly related to the radiocontrast medium, perhaps due to an idiosyncratic adverse reaction resulting in disruption of the hepatocyte canalicular membrane or interruption of transport pumps with subsequent intrahepatic cholestasis and jaundice [31, 32].

Findings of patients without short-term improvement after EST, and the occurrence of an infection with ESBL, acute necrotizing pancreatitis and biliary peritonitis within 3 to 10 months after EST revealed that EST can be of no help or has the possibility to harm the patient in the short or long run. The indication for EST should be the result of a thorough diagnostic work up of the individual patient suspect for a biliary tract disorder to rule out hepatic or pancreatic diseases requiring different therapeutic approaches. The study revealed that ERC has some advantages over US in visualizing pathologies of the biliary ductal system, but it is technically demanding and with some risk of complications. MRCP is a less invasive alternative to diagnostic ERC since it was shown to have good accuracy for the diagnosis of hepatobiliary and pancreatic duct disorders in dogs [4]. MRCP could be a beneficial imaging tool for selecting patients that require ERC and subsequent EST. MRCP does not only help to visualize the biliary and pancreatic duct systems but also the surrounding soft tissues which is impossible with ERCP. As established in human medicine, endoscopic treatment should include biliary stenting when diagnosing distal or long CBD strictures by MRCP or ERC, especially with malignant lesions that are not eligible for open surgery [33]. Endoscopic CBD stenting has been proven possible in dogs [10]. It is very likely that the establishment of MRCP in the diagnostic work up of dogs with suspected disorders of the biliary duct system could give a better indication and a more successful outcome of endoscopic interventions [4].

There are some limitations in the present study. The retrospective nature of the study caused lack of systematic reports for US and ERC/ERCP and EST. The US reports were written for clinical purposes and by different veterinary radiologists. Lack of systematic collection of laboratory, ultrasonographic and clinical follow-up data after the procedures made comparison difficult in some cases. There was also a lack of histopathologic confirmation of the lesions in some patients.

## Conclusion

Findings from this study indicate that US is an important initial imaging test in the evaluation of dogs presenting with gastrointestinal signs suggestive for a disease of the hepatobiliary system. US allows very good assessment of the gallbladder but has some limitations in assessing the papillae as well as the extra- and intrahepatic biliary duct system. ERC can serve as a complementary diagnostic procedure to improve the assessment of the biliary duct system but has limitations to assess the gallbladder and is technically demanding with risks of serious complications. EST is technically possible and offers a minimally invasive technique to treat obstructive biliary diseases in dogs. It can, however, be non-successful if the indication is not set right for the individual patient or cause serious short- and long-term complications. This demands a structured diagnostic and risk-benefit–assessment approach that is currently missing. There is also a need for more advanced diagnostic imaging of the biliary duct system, such as MRCP, to improve the selection for ERC and EST. Further investigations are needed in dogs for assessing the indications of ERCP in acute conditions.

## Methods

This study is a retrospective descriptive case series. Electronic medical records of canine patients examined between November 2007 and March 2019 were searched from data archives of the Small Animal Hospital, Faculty of Veterinary Medicine, University of Helsinki. The search for patients included the terms “ERCP”, “ERC” and “Fluoroscopy”. The criteria for inclusion of dogs were a successfully performed diagnostic ERC with or without ERP, or therapeutic EST.

The clinical records, laboratory test results (serum biochemical profile: bilirubin, ALP, ALT, cPL), US findings, ERC/ERCP findings and histopathological data of the patients were reviewed. In addition, short-term (immediately after the procedure) and, if available, long-term outcomes after the therapeutic EST procedure were obtained from the medical records. All dogs were treated medically based on their clinical problems, but these treatments are beyond the scope of this article.

### Abdominal ultrasonography

Abdominal US had been performed in all patients prior to ERC/ERCP, using an iU22 Ultrasound System (Philips, Bothell, WA) for all cases except for one dog, which had been examined using an EPIQ 7 Ultrasound System (Philips, Bothell, WA). The reports written by the examining radiologist formed the material for the present study. The following aspects were included: The liver echogenicity, compared to the falciform fat, was classified as normal, hyperechoic, hypoechoic or heterogenous. The biliary system had been evaluated for the presence of prominent major papilla, presence of distended gallbladder with debris, CBD (including diameter measurement, when available), and dilated extra- and intrahepatic ducts. The CBD was considered normal when the diameter was ≤3 mm [26, 34]. Echogenicity of the pancreas had been compared to peripancreatic mesenteric fat and was classified as normal, hyperechoic, hypoechoic or heterogenous.

### Endoscopic procedures

Indications for performing ERC/ERCP comprised chronic gastrointestinal signs, abnormal laboratory findings (hyperbilirubinemia, elevated liver enzymes and elevated cPL), and abnormal US findings in the major papilla, liver, biliary system and pancreatic area. Endoscopic procedures had been performed under general anesthesia based on previously published protocols in dogs [5, 9]. Based on these protocols, conventional gastroduodenoscopy was performed by using a standard flexible videoscope on dogs in left recumbency. ERCP was performed in ventral recumbency with an 11.0-mm side-view endoscope (JF1T10, Olympus, Tokyo, Japan) in dogs > 10 kg body weight and an 7.5-mm side-view endoscope (PJF 160, Olympus, Tokyo, Japan) in dogs < 10 kg body weight. The major and minor papillae were cannulated by inserting a sphincterotome catheter into the papillae. The sphincterotome Clever Cut 3 V (KD-V431M-0720, Olympus, Tokyo, Japan) was used for dogs > 10 kg body weight and the sphincterotome REF OE1051615GW (Endoflex GmbH, Voerde, Germany) for dogs < 10 kg body weight. The following aspects were included in the endoscopic and ERC/ERCP evaluation: prominent or stenotic major or minor papilla; abnormal, narrowed or dilated CBD based on reported normal range at different sites of the duct [5]; abnormal gallbladder filling; dilated extrahepatic ducts; dilated or abnormal filling of the intrahepatic ducts; and abnormal pancreatic duct. For contrast studies of both ductal systems, the sphincterotome catheter was filled with iodine contrast medium (Iomeprol 300, Bracco-Byk Gulden, Konstanz, Germany) before the procedure. In cases of papillary stenosis or blocked bile flow from the major papilla, EST was performed by the respective sphincterotome supported by a guidewire. For dogs > 10 kg the guidewire VisiGlide G-240-2545S (Olympus, Tokyo, Japan) was used and for dogs < 10 kg the guidewire Stripe-Guide REF 21525400 (Endoflex GmbH, Voerde, Germany). For EST of the major papilla, the tip of the sphincterotome with its diathermy wire was inserted into the papilla and bowed to bring the diathermy wire into cutting position. Then the papilla was cut open electrosurgically in steps using an electrosurgical generator (ESG-100, Olympus, Tokyo, Japan) until the bowed sphincterotome slid easily through the orifice.

In case the procedures had been performed more than once on the same animal, only the first session of each procedure was included when reporting the success rate of the procedures. However, all repeated procedures and their outcomes are also reported here.

### Histopathological examination

Samples of liver, pancreas and/or duodenum, obtained either as endo- or laparoscopic biopsies, or at laparotomy, or autopsy, were available as hematoxylin-eosin stained histological sections of formalin-fixed, paraffin-embedded tissue in 14 of 15 dogs. These samples were re-examined for the present study.

## Data Availability

The datasets used and/or analysed during the current study are available from the corresponding author on reasonable request.

## Abbreviations

US: Ultrasonography
ERCP: Endoscopic retrograde cholangiopancreatography
ERC: Endoscopic retrograde cholangiography
ERP: Endoscopic retrograde pancreatography
EST: Endoscopic sphincterotomy
MRCP: Magnetic resonance cholangiopancreatography
ALP: Alkaline phosphatase
ALT: Alanine aminotransferase
cPL: Canine pancreatic lipase
CBD: Common bile duct
ESBL: Extended spectrum beta-lactamase
